# Brain-derived blood biomarkers in multiple sclerosis—current trends and beyond

**DOI:** 10.3389/fimmu.2025.1569503

**Published:** 2025-06-16

**Authors:** Shamundeeswari Anandan, Karina Maciak, Regina Breinbauer, Sepideh Mostafavi, Christopher Elnan Kvistad, Oivind Torkildsen, Kjell-Morten Myhr

**Affiliations:** ^1^ Department of Clinical Medicine, University of Bergen, Bergen, Norway; ^2^ Neuro-SysMed, Department of Neurology, Haukeland University Hospital, Bergen, Norway; ^3^ Department of General Biochemistry, Faculty of Biology and Environmental Protection, University of Lodz, Lodz, Poland; ^4^ Faculty of Medicine, Friedrich-Alexander-University Erlangen-Nuremberg (FAU), Erlangen, Germany

**Keywords:** multiple sclerosis (MS), cerebrospinal fluid (CSF), brain-derived blood biomarkers, extracellular vesicles (EVs), magnetic resonance imaging (MRI)

## Abstract

Multiple sclerosis (MS) is a chronic inflammatory and neurodegenerative disease of the nervous system and a main cause of neurological disability in young adults. Most disease-modifying therapies are administrated as long-term maintenance therapies and may, thereby, increase the risk of infections and other immune-mediated side effects. In the last years, several cerebrospinal fluid and soluble blood biomarkers have been suggested as potential key tools for diagnosis, prognosis, and treatment monitoring of MS. Recently, the specific ability of brain-derived blood extracellular vesicles (EVs) that cross the blood-brain barrier into the bloodstream, reflecting the current immune status of the central nervous system, has kindled interest as potential biomarkers. In this review, we discuss the current trends of clinical brain-derived blood biomarkers, with a special focus on the emerging role of brain-derived blood EVs in MS.

## 1 Introduction

Almost 3 million people worldwide are affected by multiple sclerosis (MS), an immune-mediated inflammatory and degenerative disease of the central nervous system (CNS) (1). From a clinical perspective, MS is highly heterogeneous with most patients (85%–90%) experiencing an initial relapsing-remitting course (RRMS) marked by episodic inflammation and, if not treated effectively, followed by a secondary progressive (SPMS) phase, associated with gradual increasing disability (2). Epidemiological data suggest that Epstein–Barr virus is a prerequisite for developing MS, but the underlying pathogenic mechanisms are still unclear (3, 4).

The MS diagnosis relies on the combination of clinical and paraclinical findings, with no single definitive diagnostic test available (5). Currently, it is essential to determine inflammatory immune-mediated damage affecting at least two distinct regions (dissemination in space) of the CNS at varied time points (dissemination in time) to establish an MS diagnosis. Since the incorporation in the diagnostic criteria (1983), magnetic resonance imaging (MRI) of the brain and spinal cord holds a pivotal role in the diagnostic process. In addition, cerebrospinal fluid (CSF) analysis detecting intrathecal immunoglobulin G (IgG) synthesis was highlighted in the update of the diagnostic criteria of MS in 2017 (5).

Recent advancements have shed light on detecting brain-derived proteins at remarkably low concentrations in blood, paving the way for the exploration of early blood-based biomarkers in MS (6). Specific markers of immunopathological processes including neuroaxonal damage [neurofilament light chain (NfL)] and astrocyte activation [glial fibrillary acidic protein (GFAP)] are already rapidly emerging (7, 8). Extracellular vesicles (EVs) are defined as membrane-bound particles, released from virtually all cell types, with a sophisticated sorting mechanism of their cargo inclusive of lipids, proteins, and nucleic acids, in addition to carrying specific membrane proteins, mainly reflecting their donor cell. This peculiarity, plus their ability to cross the blood-brain barrier (BBB) into the blood stream, increased stability, and involvement in the regulation of both the immune system and CNS homeostasis, features brain-derived blood EVs, as improved biomarkers in CNS diseases, including MS (9–12). This review aims to summarize the current CSF and blood biomarkers in MS, discussing the unmet needs and future perspectives.

## 2 MS pathogenesis and fluid biomarkers

**Figure 1:**
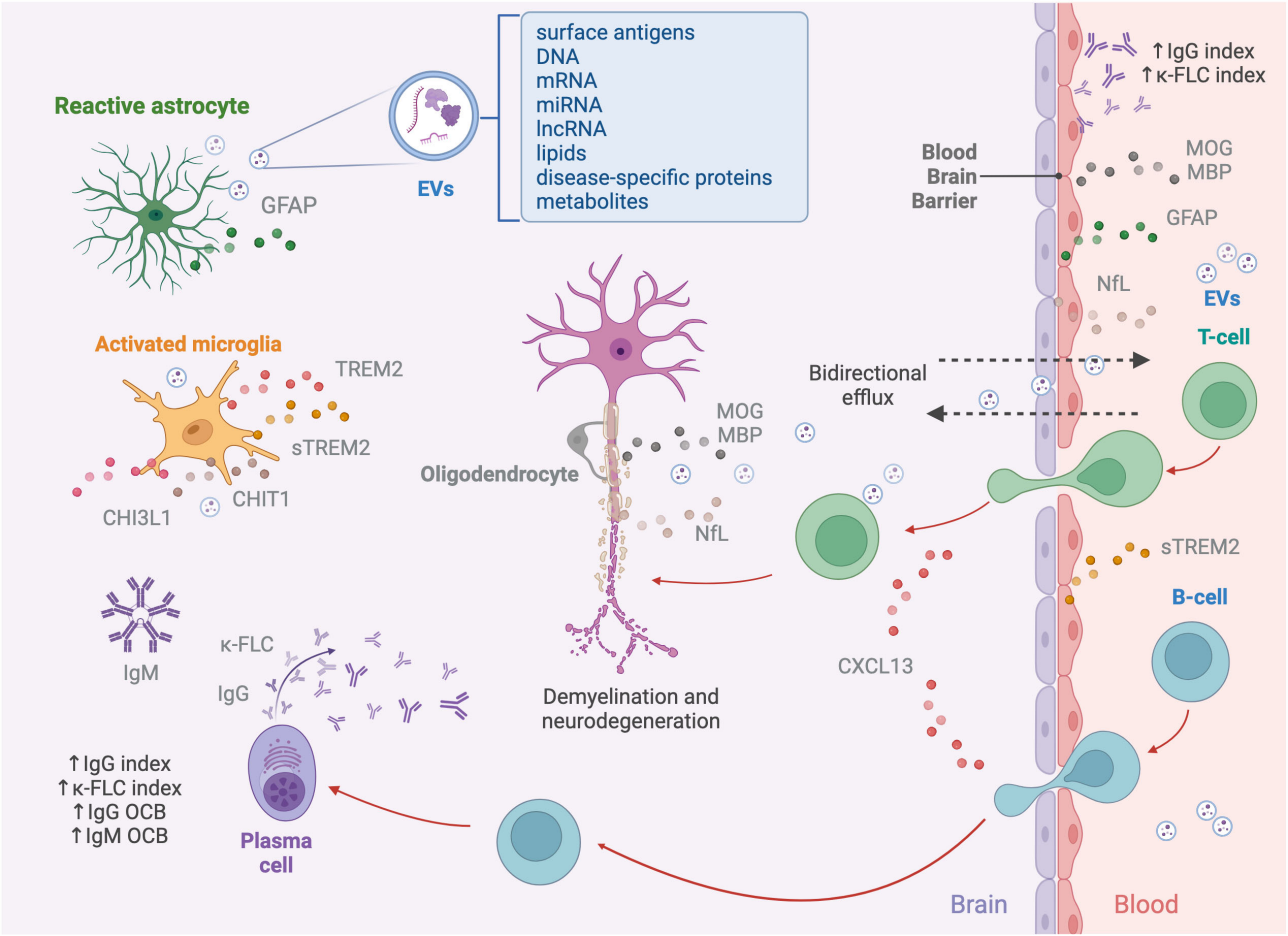
Pathophysiology of multiple sclerosis and associated biomarkers. The pathogenesis of MS begins with immune cells, including macrophages, autoreactive T cells targeting myelin, B cells, and plasma cells, infiltrating the CNS through a dysfunctional BBB. Lymphocyte recruitment is mediated by chemokines like CXCL13, which specifically attracts B cells. Within the CNS, T and B cells interact, to amplify the immune response, with T cells secreting cytokines and B cells acting as APCs. Activated B cells differentiate into plasma cells, producing immunoglobulins, including IgG and IgM and releasing κ-FLCs. The ongoing neuroinflammation leads to demyelination, axonal damage, and neurodegeneration, marked by NfL, which is released into the interstitial space, CSF, and bloodstream as a result of axonal injury, along with MOG and MBP, major proteins of the myelin sheet of oligodendrocytes. Resident immune cells within the CNS, such as microglia and astrocytes, contribute to the disruption of axonal integrity and synaptic function. While activated, microglia and astrocytes release various mediators into the CSF, including sTREM2, CHIT1, and CHI3L1. Additionally, astrocyte damage results in the release of structural proteins, such as GFAP, into the CSF and bloodstream. Another source of biomarkers reflecting pathological processes occurring in the CNS are EVs. These nanovesicles, secreted by various cell types, including neurons, astrocytes, microglia, and oligodendrocytes, carry a diverse molecular cargo, such as surface antigens, DNA, mRNA, miRNA, lncRNA, lipids, disease-specific proteins, and metabolites, acting as mediators of intercellular communication. The bidirectional efflux of EVs and soluble biomarkers across the compromised BBB enables their detection in peripheral fluids.

In the early stages of MS, the recurrent invasion of T and B cells in the brain and spinal cord drives a cascade of pathophysiological processes within the CNS (13). Several fluid biomarkers have emerged as effective indicators of this complex interaction, which contributes to the diverse clinical manifestations observed in the disease (14). Early episodes of acute focal inflammation, demyelination, and axonal damage, driven by infiltrating immune cells (macrophages, CD8^+^ T cells, CD4^+^ T cells, B cells, and plasma cells), could be typically detected through conventional MRI, showing new lesions in T2-weighted and/or T1-weighted gadolinium enhancing lesions (15, 16). Infiltrating immune cells are attracted to the CNS by several chemotactic factors such as chemokine (C-X-C motif) ligand 13 (CXCL13) for B cells (**Figure 1**) (17).

Invading T and B cells closely interact within the CNS (16, 17). In contrast to T cells, the immune pathways involving B cell activation have, so far, served as the most robust fluid biomarkers for MS. Mature plasma cells secrete IgG and IgM antibodies intrathecally, also leading to release of free light chains (due to a mismatch between immunoglobulin light- and heavy-chain synthesis) (18, 19). This inflammatory process results in axonal damage and release of neuronal markers like NfL (20). Over time, there is worsening of disability and accumulation of neurological deficits in the absence of concurrent relapses defined as “progression independent of relapse activity” (PIRA) (21). Underlying mechanism driving PIRA is increasingly understood as a pathophysiological continuum of the early “relapsing” phase driven by a chronic “smouldering” inflammatory process compartmentalized within the CNS, characterized by innate immune cells and astrocytes (22). Recent studies on positron emission tomography (PET) employing radioligands for innate immunity activation assessment have revealed an interestingly high prevalence of smouldering component in MS lesions (23). Chronically active MS lesions are slowly expanding over time or as paramagnetic rim lesions, expressing a dense network of activated iron-laden microglia/macrophages (24). Activated microglia and astrocytes release various mediators into the CSF, such as soluble triggering receptor expressed on myeloid cells 2 (sTREM2), chitinase 1 (CHIT1), chitinase-3–like protein 1 (CHI3L1), and GFAP, impacting axon, synaptic integrity, and function (25–30).

The critical role of the complement system in MS is underlined with the complement and Ig deposition across all areas of demyelination regardless of the plaque subtype, including complement-mediated myelin phagocytosis implying its importance once the disease is established. In progressive MS and long-standing disease patients, white matter plaques were consistently positive for complement proteins (C3, factor B, and C1q), regulators (factor H, C1inh, and clusterin) and activation products [C3b, iC3b, C4d, and terminal complement complex (TCC)] providing evidence that, once established, progression of inflammation in MS may not rely on infiltrating cells but rather on innate immune mechanisms including complement activation (31, 32).

EVs are pivotal in the intricate communication of neurons and glial cells of the CNS system holding neuroprotective and homeostatic effects but may have detrimental effects under pathological conditions (33, 34). EVs derived from T cells containing chemokine CCL5 and arachidonic acid can increase the expression of intercellular adhesion molecule 1 (ICAM-1) on endothelial cells and of Mac-1 on monocytes, contributing to the dysfunction of the BBB, leading to immune infiltration, a characteristic of MS pathogenesis (35–37). Dendritic cell (DCs) derived EVs carry cell surface molecules like major histocompatibility complex (MHC), ICAM-1, and other costimulatory molecules, which could aid in T-cell activation (38). EVs from activated microglia express pro-inflammatory mediators (Tumor Necrosis Factor-alpha (TNF-α) and Interleukin-1 (IL-1)) exhibiting a distinct proteomic profile enforcing inflammatory stimuli throughout the CNS (39). Recent studies show the role of astrocyte-derived EVs in the regulation of T-cell secretion and biomarker utility of myelin basic protein (MBP) and myelin oligodendrocyte glycoprotein (MOG) content in oligodendrocytes-derived EVs (40). Most immune cell–derived EVs seem to be significantly higher in treatment naïve relapsing MS patients with low disability, and their functions might depend on the physiological environment, despite limited changes in circulating immune cells (33).

## 3 MS fluid biomarkers—current trends and beyond

The diagnostic criterion for MS underscores the importance of both MRI and biofluid biomarkers emphasizing the pivotal role of accurate diagnosis, prognosis, and treatment response in the management of the disease (5). In addition to advancements in MRI techniques (7-T MRI, PET, magnetization transfer imaging, diffusion tensor imaging, and myelin water imaging), integrating biofluid biomarkers would be beneficial because of their ability to directly reflect the pathophysiological processes involved in the MS disease course (41). Cumulative evidence shows that the blood-based biomarker sNfL can predict relapses in relapsing MS patients, whereas CSF IgM oligoclonal bands, CHI3L1, and GFAP seem to be associated with a more progressive phenotype. Different aspects of microglial involvement (CHIT1 and sTREM2), astroglia pathology (CHI3L1 and GFAP), B-cell–related pathology (CXCL13), and neuroaxonal damage (sNfL) have been evaluated in several studies aiding in classifying MS disease activity (**Table 1**) (25–30). Brain-derived blood EVs (L1CAM, MOG, and GLAST) serve as potential windows into the CNS reflecting the underlying MS-related pathophysiology (**Table 1**) (33).

**Table 1:** Summary of fluid biomarkers in multiple sclerosis.

Marker	Source	Measurement methods	Clinical significance and utility	Prognostic potential	Specificity to MS	Limitations	References
Validated and completely introduced into clinical practice							
IgG OCB	CSF	Isoelectric focusing with specific IgG staining	Indicates intrathecal IgG synthesis; evidence of CNS immune activity; high sensitivity for MS diagnosis and validated biomarker in clinical utility	Predicts CIS to MS conversion; linked to disability progression	Limitation: present in other inflammatory/infectious neurological conditions	Time-consuming, qualitative method	(43–47)
IgG index	CSF and serum	Calculated as (IgG in CSF/IgG in serum)/(albumin in CSF/albumin in serum)	Measures intrathecal IgG synthesis; assesses blood-CSF barrier function	Limited; weak correlation with MS severity but linked to future disability worsening	Limitation: affected by other CNS conditions	Low sensitivity for MS diagnosis	(43–46)
κ-FLC	CSF and serum	Nephelometry, turbidimetry, κ-FLC index	Less expensive, faster quantitative alternative to OCB; detects intrathecal inflammation	Limited; predicts early relapses and disease activity in MS and enables risk stratification of disease activity in OCB-positive MS patients but still not widely validated in clinical practice	Moderate; approximately 90% diagnostic sensitivity and specificity for distinguishing MS from other neurological disorders; not exclusive to MS	Elevated in other conditions with intrathecal Ig synthesis; includes IgA and IgM (not limited to IgG)	(48–50)
Validated and not completely introduced into clinical practice							
NfL	CSF and blood (serum, plasma)	Immunoassays (e.g., ELISA); ultrasensitive immunoassays (e.g., Simoa); automated assays (e.g., Lumipulse^®^)	Reflect severity of axonal damage; elevated in RRMS and progressive MS; normalizes post-treatment	Predicts CIS to MS conversion, relapses, EDSS worsening, and brain atrophy; elevated in serum before the onset of clinical symptoms; strong marker for tissue destruction and treatment efficacy	Moderate; specific for neuronal damage but not for a disease; elevated in other neurodegenerative disorders (e.g., Alzheimer’s, traumatic brain injury)	Serum levels influenced by age and weight (can be corrected by z-score normalization); threshold values for treatment success and disease reactivation need standardization	(51–56)
Partially validated and not introduced into clinical practice							
IgM OCB	CSF	IgM index or non-linear formulas; immunoblotting; isoelectric focusing	Detects intrathecal IgM synthesis; linked to highly inflammatory RRMS and a subset of PPMS patients	Predicts shorter time to relapse and higher relapse rates; associated with disability progression and more aggressive PPMS	Moderate; found in approximately 40% of MS cases and also in other CNS conditions	Technical challenges in detection due to the high molecular weight of IgM; limited data compared to IgG OCB	(57–61)
CXCL13	CSF, serum	Immunoassays (e.g., ELISA), CXCL13 index	Elevated in early active and progressive MS; correlates with gadolinium-enhancing lesions, B-cell counts, IgG levels, κ-FLC index, relapse rate, and disease activity	Predicts CIS to MS conversion; monitors response to corticosteroids and long-term DMTs	High in CSF; independent of BBB dysfunction; undetectable in non-inflammatory controls; limitation in serum; elevated in other conditions like systemic autoimmune, inflammatory, infectious, and neoplastic diseases	Limited utility in serum—diagnostically irrelevant due to lack of CSF correlation and low specificity	(17, 62–65)
CHI3L1	CSF, serum	Immunoassays (e.g., ELISA)	Elevated in progressive MS; decreased during acute relapses compared to remission; unrelated to gadolinium lesions	Predicts CIS to MS conversion; correlates with disease progression in PPMS	Limitation: serum levels not significantly different between MS and healthy controls	Poor CSF-serum correlation; lacks specificity due to broad expression in other tissues beyond the CNS	(62, 66–69)
CHIT1	CSF, brain tissue (post-mortem)	Immunoassays (e.g., ELISA); RNA analysis in white matter tissue	Specific to microglial activation; correlates with neuronal injury (NfL) and disease activity at follow-up (up to 6 years post-diagnosis); upregulated in chronic active lesions of MS	Predicts long-term disease activity and progression; CHIT1 RNA expression differentiates chronic active lesions from chronic inactive lesions	High specificity for chronic active lesions in MS	Limited longitudinal data; needs further validation	(70–73)
sTREM2	CSF and blood (serum, plasma)	Immunoassays (e.g., ELISA)	Elevated in MS; linked to microglial activity; normalizes with natalizumab; partially reduced by mitoxantrone	Moderate correlation with EDSS and MS severity score; lack of strong correlation with other clinical measures	Limitation: elevated in other inflammatory neurological conditions	Insufficient data; weak serum-CSF correlation; not reliable as a blood biomarker	(74–78)
GFAP	CSF and blood (serum)	Immunoassays (e.g., ELISA)	Indicates astrocyte activity; reflects neuroinflammation; elevated in RRMS relapses, progressive MS; correlates with brain atrophy	Predicts disability progression in both active and non-active MS; elevated levels post-treatment indicate progression	Moderate; elevated in MS and NMOSD (predicts activity in NMOSD remission)	Labile in CSF; highly sensitive to freeze-thaw cycles; serum levels influenced by age; requires standardization for comparisons across MS subtypes	(79–83)
Not validated and not introduced into clinical practice							
CD62p^+^ EVs	Plasma	Flow cytometry	Elevated in MS vs. HC	Reflects platelet activation and monocyte interaction with damaged endothelium	Low; common in other thrombosis-related or inflammatory conditions	Overlap with other conditions	(84)
CD61^+^ EVs, CD14^+^ EVs, CD45^+^ EVs	Plasma	Flow cytometry	Elevated CD61+ EVs in untreated MS vs. HC Elevated CD61+, CD14+, and CD45+ EVs in RRMS vs. HC and SPMS	Indicates platelet activation, monocyte, and leukocyte interaction with damaged endothelium	Low; signify broader immune activation rather than MS-specific inflammation	Limited specificity for MS pathology	(85)
MOG	Serum EVs	Western blotting, ELISA	Elevated MOG EV content in RRMS patients in relapse and SPMS vs. HC	Monitors disease activity	Moderate; marker implicated in other CNS autoimmune disorders	Cross-reactivity in assays	(86, 87)
TLR3 and TLR4	Serum EVs	ELISA	Decreased TLR3 and elevated TLR4 in RRMS EVs vs. HC	Suggests altered innate immune signaling	Low; TLR expression changes occur in other autoimmune and inflammatory conditions	Requires further validation in larger cohorts	(88, 89)
Synaptopodin and synaptophysin (NEVs), complement components (AEVs)	Plasma L1CAM^+^ NEVs, plasma GLAST^+^ AEVs	ELISA, Luminex^®^	Decreased synaptopodin and synaptophysin in NEVs in MS vs. HC Elevated C1q, C3, C3b/iC3b, C5, C5a, factor H in AEVs in MS vs. HC Strong inverse correlations between both types of biomarkers in MS patients	Indicates synaptic loss and complement activation	Moderate; synaptic and complement markers are also observed in neurodegenerative diseases	Complexity in distinguishing source biomarkers	(90–92)
Absence of CD3 and CD41; presence of CD31, CD105, and CD144	Plasma EVs	Flow cytometry	Elevated concentration of CNS endothelial-derived EV in active vs. stable MS and HC	Reflects BBB permeability and active disease	Moderate; endothelial-derived markers are seen in broader CNS pathologies, reducing specificity	Limited application outside severe cases	(93)
MBP	Serum EVs	ELISA	Elevated in CIS, RRMS, and PPMS vs. HC Elevated in PPMS vs. RRMS and CIS	Correlates with EDSS and MSSS Predicts disease subtype	High; marker strongly linked to demyelination, which is a hallmark of MS	Might be not cost-effective	(40, 86, 94)
EVs concentration	Plasma EVs	NTA	Increased after 5 h of treatment with fingolimod vs. pre-treatment	Monitors treatment response	Low; observed in other conditions involving immune activation	Requires specific equipment	(95)
IB4^+^ EVs concentration	CSF	Flow cytometry	Increased in RRMS and CIS vs. HC	Reflects microglia/macrophage activation	Moderate; microglial activation is a common feature in other neuroinflammatory conditions	Limited EVs concentration in CSF	(96)
EVs concentration, CCR3^+^/CCR5^+^ EVs, CD4^+^/CCR3^+^ EVs, CD4^+^/CCR5^+^	CSF	Flow cytometry	Increased EVs in clinical relapse vs. remission Increased CCR3+/CCR5+ EVs, CD4+/CCR3+ EVs, and CD4+/CCR5+ EVs in patients with gadolinium-enhanced MR lesions	Identifies different MS phases	High; associated with active MS lesion pathology	Requires specialized equipment	(97)

MS, multiple sclerosis; HC, healthy controls; EV, extracellular vesicles; RRMS, relapsing-remitting MS; SPMS, secondary progressive MS; PPMS, primary progressive MS; CIS, clinically isolated syndrome; BBB, blood-brain barrier; EDSS, expanded disability status scale; MSSS, MS severity score; CSF, cerebrospinal fluid; CNS, central nervous system; OCB, oligoclonal bands; κ-FLC, kappa-free light chains; NGS, next-generation sequencing; NMOSD, neuromyelitis optica spectrum disorder; TLR, Toll-like receptor; NTA, nanoparticle tracking analysis; NEVs, neuron-derived extracellular vesicles; AEVs, astrocyte-derived extracellular vesicles; MOG, myelin oligodendrocyte glycoprotein; MBP, myelin basic protein; IFN-β, interferon-beta.

Certain limitations of the emerging fluid biomarkers intrude their clinical transition. For example, NfL is a promising biomarker but with limited diagnostic use due to its unspecific increase in the blood connected to several neurological conditions (42). EVs hold potential as biomarkers; however, existing knowledge gaps in terms of EVs biology, biodistribution, and assay standardization are yet to be fully elucidated (33). Although MS fluid biomarkers hold a promising frontier, addressing standardization, data validation, and accessibility are key in resolving ongoing challenges. Composite scoring with integrated clinical and MRI metrics [e.g., the MAGNIMS score or no evidence of disease activity 3 (NEDA-3) and NEDA-4] and multimodal biomarker profiling (CSF and blood-based biomarkers with neuroimaging) may be a way forward in MS management (41). Furthermore, artificial intelligence (automated lesion detection and improved diagnostic accuracy) holds transformative potential in enhancing clinical decision-making.

In conclusion, despite the limitations, the recent advances within the field hold a promising frontier, giving a paradigm shift from the conventional CSF (oligoclonal banding) analysis to a new era of brain-derived blood biomarkers (NfL, GFAP, and EVs), enabling improved longitudinal disease monitoring and personalized treatment.

## References

[1] Walton C King R Rechtman L Kaye W Leray E Marrie RA. Rising prevalence of multiple sclerosis worldwide: Insights from the Atlas of MS, third edition. Mult Scler. (2020) 26:1816–21. 10.1177/1352458520970841 PMC7720355 33174475

[2] Thompson AJ Baranzini SE Geurts J Hemmer B Ciccarelli O. Multiple sclerosis. Lancet. (2018) 391:1622–36. 10.1016/S0140-6736(18)30481-1 29576504

[3] Fernández-Fournier M López-Molina M Torres Iglesias G Botella L Chamorro B Laso-García F. Antibody content against epstein–barr virus in blood extracellular vesicles correlates with disease activity and brain volume in patients with relapsing–remitting multiple sclerosis. Int J Mol Sci. (2023) 24:14192. 37762495 10.3390/ijms241814192 PMC10531815

[4] Mrad MF Saba ES Nakib L Khoury SJ. Exosomes from subjects with multiple sclerosis express EBV-derived proteins and activate monocyte-derived macrophages. Neurol Neuroimmunology Neuroinflammation. (2021) 8:e1004. 34006621 10.1212/NXI.0000000000001004 PMC8130999

[5] Thompson AJ Banwell BL Barkhof F Carroll WM Coetzee T Comi G. Diagnosis of multiple sclerosis: 2017 revisions of the McDonald criteria. Lancet Neurology. (2018) 17:162–73. 10.1016/S1474-4422(17)30470-2 29275977

[6] Teunissen CE Kimble L Bayoumy S Bolsewig K Burtscher F Coppens S. Methods to discover and validate biofluid-based biomarkers in neurodegenerative dementias. Mol Cell Proteomics. (2023) 22:100629. 37557955 10.1016/j.mcpro.2023.100629 PMC10594029

[7] Meier S Willemse EAJ Schaedelin S Oechtering J Lorscheider J Melie-Garcia L. Serum glial fibrillary acidic protein compared with neurofilament light chain as a biomarker for disease progression in multiple sclerosis. JAMA Neurology. (2023) 80:287–97. 10.1001/jamaneurol.2022.5250 PMC10011932 36745446

[8] Di Filippo M Gaetani L Centonze D Hegen H Kuhle J Teunissen CE. Fluid biomarkers in multiple sclerosis: from current to future applications. Lancet Regional Health – Europe. (2024) 44. 10.1016/j.lanepe.2024.101009 PMC11496979 39444698

[9] Gurunathan S Kang MH Jeyaraj M Qasim M Kim JH. Review of the isolation, characterization, biological function, and multifarious therapeutic approaches of exosomes. Cells. (2019) 8. 10.3390/cells8040307 PMC6523673 30987213

[10] Huo L Du X Li X Liu S Xu Y. The emerging role of neural cell-derived exosomes in intercellular communication in health and neurodegenerative diseases. Front Neurosci. (2021) 15. 10.3389/fnins.2021.738442 PMC8438217 34531720

[11] Mycko MP Baranzini SE. microRNA and exosome profiling in multiple sclerosis. Mult Scler. (2020) 26:599–604. 31965891 10.1177/1352458519879303 PMC7160025

[12] Hornung S Dutta S Bitan G. CNS-derived blood exosomes as a promising source of biomarkers: opportunities and challenges. Front Mol Neurosci. (2020) 13. 10.3389/fnmol.2020.00038 PMC7096580 32265650

[13] Filippi M Bar-Or A Piehl F Preziosa P Solari A Vukusic S. Multiple sclerosis. Nat Rev Dis Primers. (2018) 4:43. 30410033 10.1038/s41572-018-0041-4

[14] Engelhardt B Comabella M Chan A. Multiple sclerosis: Immunopathological heterogeneity and its implications. Eur J Immunol. (2022) 52:869–81. 10.1002/eji.202149757 PMC9324211 35476319

[15] Rocca MA Preziosa P Barkhof F Brownlee W Calabrese M De Stefano N. Current and future role of MRI in the diagnosis and prognosis of multiple sclerosis. Lancet Regional Health – Europe. (2024) 44. 10.1016/j.lanepe.2024.100978 PMC11496980 39444702

[16] Dendrou CA Fugger L Friese MA. Immunopathology of multiple sclerosis. Nat Rev Immunol. (2015) 15:545–58. 10.1038/nri3871 26250739

[17] Novakova L Axelsson M Malmeström C Zetterberg H Blennow K Svenningsson A. NFL and CXCL13 may reveal disease activity in clinically and radiologically stable MS. Multiple Sclerosis Related Disord. (2020) 46:102463. 10.1016/j.msard.2020.102463 32862040

[18] Hegen H Walde J Berek K Arrambide G Gnanapavan S Kaplan B. Cerebrospinal fluid kappa free light chains for the diagnosis of multiple sclerosis: A systematic review and meta-analysis. Multiple Sclerosis J. (2022) 29:169–81. 10.1177/13524585221134213 PMC9925892 36453167

[19] Hegen H Arrambide G Gnanapavan S Kaplan B Khalil M Saadeh R. Cerebrospinal fluid kappa free light chains for the diagnosis of multiple sclerosis: A consensus statement. Multiple Sclerosis J. (2022) 29:182–95. 10.1177/13524585221134217 PMC9925908 36527368

[20] Gaetani L Blennow K Calabresi P Di Filippo M Parnetti L Zetterberg H. Neurofilament light chain as a biomarker in neurological disorders. J Neurology Neurosurg Psychiatry. (2019) 90:870–81. 10.1136/jnnp-2018-320106 30967444

[21] Kappos L Wolinsky JS Giovannoni G Arnold DL Wang Q Bernasconi C. Contribution of relapse-independent progression vs relapse-associated worsening to overall confirmed disability accumulation in typical relapsing multiple sclerosis in a pooled analysis of 2 randomized clinical trials. JAMA Neurology. (2020) 77:1132–40. 10.1001/jamaneurol.2020.1568 PMC7281382 32511687

[22] Tur C Carbonell-Mirabent P Cobo-Calvo Á Otero-Romero S Arrambide G Midaglia L. Association of early progression independent of relapse activity with long-term disability after a first demyelinating event in multiple sclerosis. JAMA Neurology. (2023) 80:151–60. 10.1001/jamaneurol.2022.4655 PMC9856884 36534392

[23] Hamzaoui M Garcia J Boffa G Lazzarotto A Absinta M Ricigliano VAG. Positron emission tomography with [F]-DPA-714 unveils a smoldering component in most multiple sclerosis lesions which drives disease progression. Ann Neurology. (2023) 94:366–83. 10.1002/ana.26657 37039158

[24] Jäckle K Zeis T Schaeren-Wiemers N Junker A van der Meer F Kramann N. Molecular signature of slowly expanding lesions in progressive multiple sclerosis. Brain. (2020) 143:2073–88. 10.1093/brain/awaa158 32577755

[25] Hinsinger G Galéotti N Nabholz N Urbach S Rigau V Demattei C. Chitinase 3-like proteins as diagnostic and prognostic biomarkers of multiple sclerosis. Multiple Sclerosis J. (2015) 21:1251–61. 10.1177/1352458514561906 25698171

[26] Cantó E Tintoré M Villar LM Costa C Nurtdinov R Álvarez-Cermeño JC. Chitinase 3-like 1: prognostic biomarker in clinically isolated syndromes. Brain. (2015) 138:918–31. 10.1093/brain/awv017 25688078

[27] Steinacker P Verde F Fang L Feneberg E Oeckl P Roeber S. Chitotriosidase (CHIT1) is increased in microglia and macrophages in spinal cord of amyotrophic lateral sclerosis and cerebrospinal fluid levels correlate with disease severity and progression. J Neurology Neurosurg Psychiatry. (2018) 89:239–47. 10.1136/jnnp-2017-317138 29142138

[28] Filipello F Goldsbury C You SF Locca A Karch CM Piccio L. Soluble TREM2: Innocent bystander or active player in neurological diseases? Neurobiol Dis. (2022) 165:105630. 35041990 10.1016/j.nbd.2022.105630 PMC10108835

[29] Högel H Rissanen E Barro C Matilainen M Nylund M Kuhle J. Serum glial fibrillary acidic protein correlates with multiple sclerosis disease severity. Multiple Sclerosis J. (2018) 26:210–9. 10.1177/1352458518819380 30570436

[30] Abdelhak A Foschi M Abu-Rumeileh S Yue JK D’Anna L Huss A. Blood GFAP as an emerging biomarker in brain and spinal cord disorders. Nat Rev Neurology. (2022) 18:158–72. 10.1038/s41582-021-00616-3 35115728

[31] Breij EC Brink BP Veerhuis R Van den Berg C Vloet R Yan R. Homogeneity of active demyelinating lesions in established multiple sclerosis. Ann Neurology: Off J Am Neurological Assoc Child Neurol Society. (2008) 63:16–25. 10.1002/ana.21311 18232012

[32] Ingram G Loveless S Howell OW Hakobyan S Dancey B Harris CL. Complement activation in multiple sclerosis plaques: an immunohistochemical analysis. Acta neuropathologica Commun. (2014) 2:1–15. 10.1186/2051-5960-2-53 PMC4048455 24887075

[33] Pistono C Osera C Cuccia M Bergamaschi R. Roles of extracellular vesicles in multiple sclerosis: from pathogenesis to potential tools as biomarkers and therapeutics. Sclerosis. (2023) 1:91–112.

[34] Schnatz A Müller C Brahmer A Krämer-Albers EM. Extracellular Vesicles in neural cell interaction and CNS homeostasis. FASEB Bioadv. (2021) 3:577–92. 10.1096/fba.2021-00035 PMC8332475 34377954

[35] Sáenz-Cuesta M Osorio-Querejeta I Otaegui D. Extracellular vesicles in multiple sclerosis: what are they telling us? Front Cell Neurosci. (2014) 8:100. 24734004 10.3389/fncel.2014.00100 PMC3975116

[36] Barry OP Kazanietz MG Pratico D FitzGerald GA. Arachidonic acid in platelet microparticles up-regulates cyclooxygenase-2-dependent prostaglandin formation via a protein kinase C/mitogen-activated protein kinase-dependent pathway. J Biol Chem. (1999) 274:7545–56. 10.1074/jbc.274.11.7545 10066822

[37] Quandt J Dorovini-Zis K. The beta chemokines CCL4 and CCL5 enhance adhesion of specific CD4+ T cell subsets to human brain endothelial cells. J Neuropathology Exp Neurology. (2004) 63:350–62. 10.1093/jnen/63.4.350 15099025

[38] Segura E Nicco C Lombard B Véron P Raposo G Batteux F. ICAM-1 on exosomes from mature dendritic cells is critical for efficient naive T-cell priming. Blood. (2005) 106:216–23. 10.1182/blood-2005-01-0220 15790784

[39] Aires ID Ribeiro-Rodrigues T Boia R Ferreira-Rodrigues M Girão H Ambrósio AF. Microglial extracellular vesicles as vehicles for neurodegeneration spreading. Biomolecules. (2021) 11:770. 34063832 10.3390/biom11060770 PMC8224033

[40] Agliardi C Guerini FR Zanzottera M Bolognesi E Picciolini S Caputo D. Myelin basic protein in oligodendrocyte-derived extracellular vesicles as a diagnostic and prognostic biomarker in multiple sclerosis: a pilot study. Int J Mol Sci. (2023) 24:894. 36614334 10.3390/ijms24010894 PMC9821098

[41] Anderhalten L Wohlrab F Paul F. Emerging MRI and biofluid biomarkers in the diagnosis and prognosis of multiple sclerosis. Lancet Regional Health – Europe. (2024) 44. 10.1016/j.lanepe.2024.101023 PMC11496965 39444705

[42] eBioMedicine. Blood biomarkers for multiple sclerosis: neurofilament light chain and beyond. eBioMedicine. (2024) 104. 10.1016/j.ebiom.2024.105214 PMC11245755 38880550

[43] Link H Huang Y-M. Oligoclonal bands in multiple sclerosis cerebrospinal fluid: An update on methodology and clinical usefulness. J Neuroimmunology. (2006) 180:17–28. 16945427 10.1016/j.jneuroim.2006.07.006

[44] McLean BN Luxton RW Thompson EJ. A study of immunoglobulin G in the cerebrospinal fluid of 1007 patients with suspected neurological disease using isoelectric focusing and the Log IgG-Index. A comparison and diagnostic applications. Brain. (1990) 113:1269–89. 10.1093/brain/113.5.1269 2245296

[45] Lunding J Midgard R Vedeler CA. Oligoclonal bands in cerebrospinal fluid:a comparative study of isoelectric focusing, agarose gel electrophoresis and IgG index. Acta Neurologica Scandinavica. (2000) 102:322–5. 10.1034/j.1600-0404.2000.102005322.x 11083510

[46] Arrambide G Espejo C Carbonell-Mirabent P Dieli-Crimi R Rodríguez-Barranco M Castillo M. The kappa free light chain index and oligoclonal bands have a similar role in the McDonald criteria. Brain. (2022) 145:3931–42. 10.1093/brain/awac220 35727945

[47] Rojas Juan I Patrucco L Cristiano E. Oligoclonal bands and MRI in clinically isolated syndromes: predicting conversion time to multiple sclerosis. J Neurol. (2010) 257:1188–91. 10.1007/s00415-010-5490-y 20157721

[48] Berek K Bsteh G Auer M Di Pauli F Grams A Milosavljevic D. Kappa-free light chains in CSF predict early multiple sclerosis disease activity. Neurol Neuroimmunology Neuroinflammation. (2021) 8:e1005. 34049994 10.1212/NXI.0000000000001005 PMC8168046

[49] Dekeyser C De Kesel P Cambron M Vanopdenbosch L Van Hijfte L Vercammen M. Inter-assay diagnostic accuracy of cerebrospinal fluid kappa free light chains for the diagnosis of multiple sclerosis. Front Immunol. (2024). 10.3389/fimmu.2024.1385231 PMC11091388 38745673

[50] Duell F Evertsson B Al Nimer F Sandin Å Olsson D Olsson T. Diagnostic accuracy of intrathecal kappa free light chains compared with OCBs in MS. Neurol Neuroimmunology Neuroinflammation. (2020) 7:e775. 32527760 10.1212/NXI.0000000000000775 PMC7309528

[51] Ning L Wang B. Neurofilament light chain in blood as a diagnostic and predictive biomarker for multiple sclerosis: A systematic review and meta-analysis. PloS One. (2022) 17:e0274565. 36103562 10.1371/journal.pone.0274565 PMC9473405

[52] Freedman MS Gnanapavan S Booth RA Calabresi PA Khalil M Kuhle J. Guidance for use of neurofilament light chain as a cerebrospinal fluid and blood biomarker in multiple sclerosis management. EBioMedicine. (2024) 101:104970. 38354532 10.1016/j.ebiom.2024.104970 PMC10875256

[53] Bäckström D Linder J Jakobson Mo S Riklund K Zetterberg H Blennow K. NfL as a biomarker for neurodegeneration and survival in Parkinson disease. Neurology. (2020) 95:e827–e38. 10.1212/WNL.0000000000010084 PMC7605503 32680941

[54] Sahrai H Norouzi A Hamzehzadeh S Majdi A Kahfi-Ghaneh R Sadigh-Eteghad S. SIMOA-based analysis of plasma NFL levels in MCI and AD patients: a systematic review and meta-analysis. BMC Neurology. (2023) 23:331. 37723414 10.1186/s12883-023-03377-2 PMC10506291

[55] Urbano T Maramotti R Tondelli M Gallingani C Carbone C Iacovino N. Comparison of serum and cerebrospinal fluid neurofilament light chain concentrations measured by ella™ and lumipulse™ in patients with cognitive impairment. Diagnostics. (2024) 14:2408. 39518375 10.3390/diagnostics14212408 PMC11544876

[56] Vecchio D Puricelli C Malucchi S Virgilio E Martire S Perga S. Serum and cerebrospinal fluid neurofilament light chains measured by SIMOA™, Ella™, and Lumipulse™ in multiple sclerosis naïve patients. Mult Scler Relat Disord. (2024) 82:105412. 38198989 10.1016/j.msard.2023.105412

[57] Magliozzi R Mazziotti V Montibeller L Pisani AI Marastoni D Tamanti A. Cerebrospinal fluid igM levels in association with inflammatory pathways in multiple sclerosis patients. Front Cell Neurosci. (2020) 14. 10.3389/fncel.2020.569827 PMC7596330 33192314

[58] Mandrioli J Sola P Bedin R Gambini M Merelli E. A multifactorial prognostic index in multiple sclerosis. J Neurology. (2008) 255:1023–31. 10.1007/s00415-008-0827-5 18535872

[59] Villar LM Casanova B Ouamara N Comabella M Jalili F Leppert D. Immunoglobulin M oligoclonal bands: biomarker of targetable inflammation in primary progressive multiple sclerosis. Ann Neurol. (2014) 76:231–40. 10.1002/ana.24190 24909126

[60] Villar LM Sádaba MC Roldán E Masjuan J González-Porqué P Villarrubia N. Intrathecal synthesis of oligoclonal IgM against myelin lipids predicts an aggressive disease course in MS. J Clin Invest. (2005) 115:187–94. 10.1172/JCI22833 PMC539201 15630459

[61] Villar LM González-Porqué P Masjuán J Alvarez-Cermeño JC Bootello A Keir G. A sensitive and reproducible method for the detection of oligoclonal IgM bands. J Immunol Methods. (2001) 258:151–5. 10.1016/s0022-1759(01)00492-6 11684132

[62] Pike SC Gilli F Pachner AR. The CXCL13 index as a predictive biomarker for activity in clinically isolated syndrome. Int J Mol Sci. (2023) 24. 10.3390/ijms241311050 PMC10341709 37446228

[63] Lucchini M De Arcangelis V Piro G Nociti V Bianco A De Fino C. CSF CXCL13 and chitinase 3-like-1 levels predict disease course in relapsing multiple sclerosis. Mol Neurobiology. (2023) 60:36–50. 10.1007/s12035-022-03060-6 PMC9758105 36215027

[64] Khademi M Kockum I Andersson ML Iacobaeus E Brundin L Sellebjerg F. Cerebrospinal fluid CXCL13 in multiple sclerosis: a suggestive prognostic marker for the disease course. Mult Scler. (2011) 17:335–43. 10.1177/1352458510389102 21135023

[65] DiSano KD Gilli F Pachner AR. Intrathecally produced CXCL13: A predictive biomarker in multiple sclerosis. Mult Scler J Exp Transl Clin. (2020) 6:2055217320981396. 33403120 10.1177/2055217320981396 PMC7747124

[66] Mohammed MS Al-Rubae'i SHN Rheima AM Al-Kazazz FF. A novel sandwich ELISA method for quantifying CHI3L1 in blood serum and cerebrospinal fluid multiple sclerosis patients using sustainable photo-irradiated zero-valence gold nanoparticles. Results Chem. (2024) 11:101856.

[67] Pérez-Miralles F Prefasi D García-Merino A Gascón-Giménez F Medrano N Castillo-Villalba J. CSF chitinase 3-like-1 association with disability of primary progressive MS. Neurol Neuroimmunol Neuroinflamm. (2020) 7. 10.1212/NXI.0000000000000815 PMC7357419 32611760

[68] Floro S Carandini T Pietroboni AM De Riz MA Scarpini E Galimberti D. Role of chitinase 3–like 1 as a biomarker in multiple sclerosis. Neurol Neuroimmunology Neuroinflammation. (2022) 9:e1164. 35534236 10.1212/NXI.0000000000001164 PMC9128043

[69] Cantó E Reverter F Morcillo-Suárez C Matesanz F Fernández O Izquierdo G. Chitinase 3-like 1 plasma levels are increased in patients with progressive forms of multiple sclerosis. Mult Scler. (2012) 18:983–90. 10.1177/1352458511433063 22183936

[70] Oldoni E Smets I Mallants K Vandebergh M Van Horebeek L Poesen K. CHIT1 at diagnosis reflects long-term multiple sclerosis disease activity. Ann Neurol. (2020) 87:633–45. 10.1002/ana.25691 PMC7187166 31997416

[71] Beliën J Swinnen S D’hondt R Verdú de Juan L Dedoncker N Matthys P. CHIT1 at diagnosis predicts faster disability progression and reflects early microglial activation in multiple sclerosis. Nat Commun. (2024) 15:5013. 38866782 10.1038/s41467-024-49312-y PMC11169395

[72] Rabin A Bello E Kumar S Zeki DA Afshari K Deshpande M. Targeted proteomics of cerebrospinal fluid in treatment naïve multiple sclerosis patients identifies immune biomarkers of clinical phenotypes. Sci Rep. (2024) 14:21793. 39294186 10.1038/s41598-024-67769-1 PMC11411093

[73] Comabella M Fernández M Martin R Rivera-Vallvé S Borrás E Chiva C. Cerebrospinal fluid chitinase 3-like 1 levels are associated with conversion to multiple sclerosis. Brain. (2010) 133:1082–93. 10.1093/brain/awq035 20237129

[74] Ioannides ZA Csurhes PA Swayne A Foubert P Aftab BT Pender MP. Correlations between macrophage/microglial activation marker sTREM-2 and measures of T-cell activation, neuroaxonal damage and disease severity in multiple sclerosis. Mult Scler J Exp Transl Clin. (2021) 7:20552173211019772. 34158970 10.1177/20552173211019772 PMC8182190

[75] Piccio L Buonsanti C Cella M Tassi I Schmidt RE Fenoglio C. Identification of soluble TREM-2 in the cerebrospinal fluid and its association with multiple sclerosis and CNS inflammation. Brain. (2008) 131:3081–91. 10.1093/brain/awn217 PMC2577803 18790823

[76] Öhrfelt A Axelsson M Malmeström C Novakova L Heslegrave A Blennow K. Soluble TREM-2 in cerebrospinal fluid from patients with multiple sclerosis treated with natalizumab or mitoxantrone. Mult Scler. (2016) 22:1587–95. 10.1177/1352458515624558 26754805

[77] Ashton NJ Suárez-Calvet M Heslegrave A Hye A Razquin C Pastor P. Plasma levels of soluble TREM2 and neurofilament light chain in TREM2 rare variant carriers. Alzheimer’s Res Ther. (2019) 11:94. 31779670 10.1186/s13195-019-0545-5 PMC6883551

[78] Cignarella F Filipello F Bollman B Cantoni C Locca A Mikesell R. TREM2 activation on microglia promotes myelin debris clearance and remyelination in a model of multiple sclerosis. Acta Neuropathol. (2020) 140:513–34. 10.1007/s00401-020-02193-z PMC7498497 32772264

[79] Sun M Liu N Xie Q Li X Sun J Wang H. A candidate biomarker of glial fibrillary acidic protein in CSF and blood in differentiating multiple sclerosis and its subtypes: A systematic review and meta-analysis. Mult Scler Relat Disord. (2021) 51:102870. 33819724 10.1016/j.msard.2021.102870

[80] Barro C Healy BC Liu Y Saxena S Paul A Polgar-Turcsanyi M. Serum GFAP and nfL levels differentiate subsequent progression and disease activity in patients with progressive multiple sclerosis. Neurol Neuroimmunol Neuroinflamm. (2023) 10. 10.1212/NXI.0000000000200052 PMC9749933 36376097

[81] Rosenstein I Nordin A Sabir H Malmeström C Blennow K Axelsson M. Association of serum glial fibrillary acidic protein with progression independent of relapse activity in multiple sclerosis. J Neurol. (2024) 271:4412–22. 10.1007/s00415-024-12389-y PMC11233378 38668889

[82] Schindler P Aktas O Ringelstein M Wildemann B Jarius S Paul F. Glial fibrillary acidic protein as a biomarker in neuromyelitis optica spectrum disorder: a current review. Expert Rev Clin Immunol. (2023) 19:71–91. 36378751 10.1080/1744666X.2023.2148657

[83] Simrén J Weninger H Brum WS Khalil S Benedet AL Blennow K. Differences between blood and cerebrospinal fluid glial fibrillary Acidic protein levels: The effect of sample stability. Alzheimers Dement. (2022) 18:1988–92. 10.1002/alz.12806 PMC9826213 36102852

[84] Sheremata WA Jy W Delgado S Minagar A McLarty J Ahn Y. Interferon-beta1a reduces plasma CD31+ endothelial microparticles (CD31+EMP) in multiple sclerosis. J Neuroinflammation. (2006) 3:23. 16952316 10.1186/1742-2094-3-23 PMC1584221

[85] Sáenz-Cuesta M Haritz I Tamara C-T Maider M-C Iñaki O-Q Alvaro P. Circulating microparticles reflect treatment effects and clinical status in multiple sclerosis. Biomarkers Med. (2014) 8:653–61. 10.2217/bmm.14.9 25123034

[86] Galazka G Mycko MP Selmaj I Raine CS Selmaj KW. Multiple sclerosis: Serum-derived exosomes express myelin proteins. Mult Scler. (2018) 24:449–58. 10.1177/1352458517696597 28273783

[87] Moseley CE Virupakshaiah A Forsthuber TG Steinman L Waubant E Zamvil SS. MOG CNS autoimmunity and MOGAD. Neurol Neuroimmunol Neuroinflamm. (2024) 11:e200275. 38996203 10.1212/NXI.0000000000200275 PMC11256982

[88] D’Anca M Fenoglio C Buccellato FR Visconte C Galimberti D Scarpini E. Extracellular vesicles in multiple sclerosis: role in the pathogenesis and potential usefulness as biomarkers and therapeutic tools. Cells. (2021) 10. 10.3390/cells10071733 PMC8303489 34359903

[89] Bhargava P Nogueras-Ortiz C Chawla S Bæk R Jørgensen MM Kapogiannis D. Altered levels of toll-like receptors in circulating extracellular vesicles in multiple sclerosis. Cells. (2019) 8. 10.3390/cells8091058 PMC6769450 31509962

[90] Bhargava P Nogueras-Ortiz C Kim S Delgado-Peraza F Calabresi PA Kapogiannis D. Synaptic and complement markers in extracellular vesicles in multiple sclerosis. Mult Scler. (2021) 27:509–18. 10.1177/1352458520924590 PMC7744427 32669030

[91] Nogueras-Ortiz CJ Eren E Yao P Calzada E Dunn C Volpert O. Single-extracellular vesicle (EV) analyses validate the use of L1 Cell Adhesion Molecule (L1CAM) as a reliable biomarker of neuron-derived EVs. J Extracell Vesicles. (2024) 13:e12459. 38868956 10.1002/jev2.12459 PMC11170079

[92] Li D Zou S Huang Z Sun C Liu G. Isolation and quantification of L1CAM-positive extracellular vesicles on a chip as a potential biomarker for Parkinson’s Disease. J Extracell Vesicles. (2024) 13:e12467. 38898558 10.1002/jev2.12467 PMC11186740

[93] Mazzucco M Mannheim W Shetty SV Linden JR. CNS endothelial derived extracellular vesicles are biomarkers of active disease in multiple sclerosis. Fluids Barriers CNS. (2022) 19:13. 35135557 10.1186/s12987-021-00299-4 PMC8822708

[94] Martinsen V Kursula P. Multiple sclerosis and myelin basic protein: insights into protein disorder and disease. Amino Acids. (2022) 54:99–109. 34889995 10.1007/s00726-021-03111-7 PMC8810476

[95] Sáenz-Cuesta M Alberro A Muñoz-Culla M Osorio-Querejeta I Fernandez-Mercado M Lopetegui I. The first dose of fingolimod affects circulating extracellular vesicles in multiple sclerosis patients. Int J Mol Sci. (2018) 19. 10.3390/ijms19082448 PMC6121302 30126230

[96] Verderio C Muzio L Turola E Bergami A Novellino L Ruffini F. Myeloid microvesicles are a marker and therapeutic target for neuroinflammation. Ann Neurol. (2012) 72:610–24. 10.1002/ana.23627 23109155

[97] Geraci F Ragonese P Barreca MM Aliotta E Mazzola MA Realmuto S. Differences in intercellular communication during clinical relapse and gadolinium-enhanced MRI in patients with relapsing remitting multiple sclerosis: A study of the composition of extracellular vesicles in cerebrospinal fluid. Front Cell Neurosci. (2018) 12:418. 30498433 10.3389/fncel.2018.00418 PMC6249419

